# Comparison of Peyton’s Four-Step Approach With the Conventional Bedside Technique in Teaching Clinical Examination Skills to Medical Students

**DOI:** 10.7759/cureus.54397

**Published:** 2024-02-18

**Authors:** Sajit Varghese, Lissa Abraham

**Affiliations:** 1 General Medicine, Pushpagiri Institute of Medical Sciences & Research Center, Thiruvalla, IND; 2 Emergency Medicine, Pushpagiri Institute of Medical Sciences & Research Center, Thiruvalla, IND

**Keywords:** peyton’s four-step approach, performance, comprehension, deconstruction, demonstration, medical students, conventional bedside teaching, bedside clinical examination skills

## Abstract

Background: Conventional bedside teaching (CBT) is an integral and classical method for imparting clinical skills to undergraduates in medical schools. It is a traditionally successful approach, especially when it comes to imparting patient-doctor relationship skills and knowledge on clinical management. Peyton’s four-step approach (PFSA) is one of the newer structured instructional approaches for teaching-learning, especially for imparting procedural and complex psychomotor skills. The present study compares the application of PFSA in teaching complex systemic examination skills to the CBT technique in teaching the same skill to MBBS students. The impact of the acquisition of this examination skill was assessed statistically to compare PFSA and CBT methodologies.

Methodology: The target population was MBBS (Bachelor of Medicine and Bachelor of Surgery) students; for this study, the phase II MBBS students were considered as the study population since they were relatively naïve to clinical bedside examination skills. Students were allotted groups and they were taught clinical skills through CBT and PFSA separately. Using the OpenEpi toolkit Version 3 open-source sample size calculator for comparing two means, the sample size was 30 students in each group. The students were assessed for their competency and were also made to fill out a feedback questionnaire to compare the two methods of education dispensing.

Results: The results of this study showed that PFSA is definitely suitable for teaching clinical examination skills. The acquisition of skills was found non-inferior to CBT while the retention of these skills was found to be equally good or even superior with PFSA than with CBT.

Conclusion: PFSA has already been proven to be a good teaching method for the acquisition of complex procedural skills. This study expands the role of PFSA in teaching clinical bedside examination skills to medical students. Further large-scale studies may clarify the learning impact and outcomes of PFSA in clinical bedside teaching.

## Introduction

Conventional bedside teaching (CBT) is an integral and classical method for imparting clinical skills to undergraduates in medical schools. It is a traditionally successful approach, especially when it comes to imparting patient-doctor relationship skills and knowledge on clinical management. However, ensuring clinical competence requires a proper and sustainable acquisition of clinical skills and knowledge [1]. CBT is poorly structured and often fails to achieve a uniform standard in the achievement of basic clinical examination skills [2]. There has been a global awakening over the last decade in terms of achieving learner-centered constructive education rather than persisting with the conventional teacher-oriented behavioristic approach [3]. Clinical skill acquisition must not be the result of learning from mimicking but from acquiring competence through observation, practice, dialogue, and feedback [4]. Peyton’s four-step approach (PFSA) is one of the newer structured instructional approaches for teaching-learning, especially for imparting procedural and complex psychomotor skills [5]. It has proved to be superior to traditional teaching methods like ‘See One, Do One, and Teach One’ for teaching surgical skills [6].

PFSA is effective even for training large groups and has been accepted by trainees of late [7]. PFSA comprises four steps, namely, 'Demonstration' wherein the trainer performs the skill at a normal speed and without additional comments, while the trainee observes. The second step is 'Deconstruction' in which the trainer repeats the skill step by step, with the necessary explanation. In the next step, the trainer explains each step and instructs the trainee, who performs the steps on the command which is 'Comprehension'. The last step is 'Performance' wherein the trainee performs the entire skill by self [8]. PFSA combines attention, performance observation, feedback, motor imagination, and two-way dialogue [9]. Initially, PFSA was devised as a one-on-one teaching-learning model but now various modifications of PFSA have been validated for small-group and large-group teaching modules [10].

Medical school teachers all over the world are now increasingly using this method to teach procedural and psychomotor skills to undergraduates [11]. It has been seen that the imparting of knowledge does not always and necessarily translate into clinical skill mastery, using the traditional bedside teaching technique [12]. The application of PFSA in teaching clinical systemic examination skills and its comparison to CBT has not been studied scientifically to date. There is a dearth of valid studies and reliable data when it comes to applying PFSA in teaching physical examination skills to undergraduates in medical schools.

Levin et al. opined that the approach for teaching clinical and procedural skills to medical undergraduates is gradually shifting towards a structured learner-centric approach [13]. Duca et al. explained the gap in the present curriculum in imparting clinical examination skills to medical students [14]. Custers in 2018 pointed out the lacunae in the CBT techniques [15]. Hence, there is a need to explore and analyze whether structured instructional pedagogies will fare better in teaching the acquisition of clinical examination skills to medical students. The literature describes PFSA as a proven and effective tool, consisting of two-way dialogue, active learning, and feedback, in imparting clinical skills to students. Skrzypek et al. (2020) proposed PFSA as a teaching tool for imparting clinical and examination skills to medical students [16]. Ramani and Leinster in 2008 explained in detail the complexities of adequately imparting clinical examination skills so that these skills are not only acquired but also effectively retained by medical students [17]. Rammell et al. in 2018 showed the importance of incorporating synchronous feedback-based methods in imparting clinical skills [18]. Schmidt and Mamede in 2015 narrated the importance of innovating structured teaching tools to impart clinical skills to students and the dearth of research in this field [19]. The present study aims to work on this research gap and analyze whether PFSA is effective in imparting the acquisition and retention of clinical examination skills to medical undergraduates.

The present study was undertaken to compare the application of PFSA in teaching complex systemic examination skills (examination of tone, elicitation of deep tendon reflex, elicitation of signs of free fluid in the abdomen) to the CBT technique in teaching the same skill to MBBS (Bachelor of Medicine and Bachelor of Surgery) students. The impact of the acquisition of this examination skill was assessed statistically to compare PFSA and CBT methodologies. One of the primary objectives of the study was also to assess the learning impact of the skill teaching technique, and this is done by an OSCE (Objective Structured Clinical Examination) [20].

## Materials and methods

Study design and setting

It was a single-center, prospective interventional study. The reporting and article preparation for the cross-sectional aspects of the study adhered to the Strengthening the Reporting of Observational Studies in Epidemiology (STROBE) recommendations. The study was conducted at the Department of General Medicine, Pushpagiri Medical College Hospital, Thiruvalla, Kerala in South India. 

Study participants

The target population was MBBS students; for this study, phase II MBBS students were considered as the study population, since they were relatively naïve to clinical bedside examination skills. The current study included undergraduate students belonging to phase II MBBS, posted under the Department of General Medicine, Pushpagiri Medical College Hospital, Thiruvalla, Kerala. Informed consent was obtained from all participants and those students who refused to participate in the study were excluded. Institutional Review Board approval was obtained before the study (No. IRB/I/15/2022) and strictly complied with.

*Sample Size and Sampling Procedure*

An independent faculty from the Department of General Medicine assigned a serial number from the sampling sequence to each of the eligible study participants and dispensed them through a simple random sampling method into two groups A and B. The particular allocating faculty had no further role in the study to avoid any confounders. Group A participants were taught the clinical examination skills (examination of tone, elicitation of deep tendon reflexes, and elicitation of signs of free fluid in the abdomen) by PFSA technique while Group B participants were taught the same skillset by CBT technique.

Based on a previous study conducted by Gradl-Dietsch et al. (2016), using the OpenEpi toolkit Version 3 open-source sample size calculator for comparing two means, with a confidence interval of 95%, power of 80%, equal sample size ratio between the two groups and considering the mean values in assessment total score in that study, a sample size of 30 participants in each group for the present study was computed [5].

Data sources and variables

Conventional Bedside Teaching

The CBT technique is traditionally the standard of teaching bedside examination and comprises an expert faculty (physician) teaching and demonstrating the clinical skills over a patient by the bedside while the undergraduate students surround the patient’s bed and observe keenly. 

Peyton’s Four-Step Approach

PFSA comprises four sequences, namely, Demonstration, Deconstruction, Comprehension, and Performance. In the PFSA technique, the expert faculty (physician) first shows the clinical skill to the observing student and then again repeats the skill slowly, explaining each step to the student. After that, in the Comprehension step, the physician performs the skill on instructional command from the student while in the last step, the student performs by self. The three clinical examination skill sets chosen for this study were examination of tone, elicitation of deep tendon reflexes, and elicitation of signs of free fluid in the abdomen. Since this was a teaching session for clinical examination skills only and did not explain the theoretical knowledge in detail, all three skill sets could be demonstrated in a single teaching session for the participating MBBS students. 

Objective Structured Clinical Examination

An OSCE is a versatile and valid tool to assess competency in clinical skills acquisition [20]. OSCE checklist consisted of three sections, of which section A dealt with the examination of tone, section B dealt with the elicitation of deep tendon jerks, and section C dealt with the elicitation of signs of free fluid in the abdomen. For a good performance rating in this OSCE, a total score of 27 or more out of 30 (combining a total out of 10 for each section) would be needed. The OSCE test could be completed within 10 minutes.

Perception Questionnaire

A perception questionnaire was also used which had six questions related to the experience of students in the teaching methods which were answered by the students on a Likert scale. This questionnaire was developed by the investigators and was duly reviewed and approved by the institutional review board. 

Procedure

Two instructors were selected for the teaching of clinical skills and they were faculties belonging to the Department of General Medicine of the institution, with a minimum experience of 1 year in a medical college setup. They were independent and only took part in dispensing the teaching of the skill, during the clinical posting hours, for a maximum period of 10 minutes. One of them trained Group A and the other Group B, exclusively, to prevent confounders and inter-faculty variability. Two evaluators were selected for the assessment of clinical skills acquisition and retention, and they were faculty members belonging to the Department of General Medicine of the institution. Instructors and evaluators were not the same persons to avoid pre-determined perceptions.

Evaluators were blinded toward the design and aims of the present study and also unaware of the allocated group of each participant. The assessment was done by an OSCE the next day (that is, after 24 hours of instructional teaching by PFSA or CBT technique). In the next week, the second assessment for evaluating retention of clinical skills comprising of a similar OSCE was done on any of the days 8th, 9th, or 10th (starting from the date of instructional teaching). Assessment 1 (on the next day of teaching) was done by one evaluator while Assessment 2 (after one week of teaching) was done by the other evaluator, thus ensuring that each participant is assessed by both the evaluators separately and consistently. The Perception Questionnaire, consisting of six statements was filled by the participant at the end of this second OSCE test.

Data collection and analysis

The data was collected via the pre-designed pro forma which included specific participant assigned serial number, participant name, gender, age, assigned group name (A or B), date of instructional teaching (by any of PFSA or CBT method), date of Assessment 1, total score in Section A (out of 10) of OSCE, total score in Section B (out of 10) of OSCE, total score in Section C (out of 10), Grand Total of OSCE (Assessment 1), Good Performance rating in first OSCE (Yes or No), date of Assessment 2, total score in Section A, B and C (each out of 10) for Assessment 2, Good Performance rating in second OSCE (Yes or No) and Perception Questionnaire completion and submission (Yes/No). The collection of data was entirely done by the investigator, who also verified and checked for any errors or missed data at the end of data collection completion of each participant (that is, after 1 week of intervention). The principal investigator ensured that there was no lag in the schedule of assessment on the next day of instructional teaching and after one week, as planned in the methodology, for any participant. 

Data was entered in a Microsoft Excel spreadsheet and then analyzed using IBM SPSS Statistics for Windows, Version 20 (Released 2011; IBM Corp., Armonk, New York, United States). For categorical variables, the Chi-square test was used to assess differences. Quantitative variables were summarized as mean with standard deviation. The differences in the means of the two groups A and B were assessed for statistical significance by the unpaired t-test. The level of significance was fixed at p<0.05 for statistical tests. 

## Results

Demographic characteristics

Table 1 summarizes the baseline demographic characteristics of the study participants. There were 30 students in each group. Both groups A and B were comparable in terms of age and gender, with no evident significant difference. 

**Table 1:** Demographic characteristics of the two groups SD= standard deviation, N = number Group A - participants were taught the clinical examination skills by the PFSA technique Group B - participants were taught the clinical examination skills by the CBT technique The differences in the means of the two groups A and B were assessed for statistical significance by unpaired t-test. The level of significance was fixed at p<0.05 for statistical tests. PFSA: Peyton's four-step approach; CBT: conventional bedside teaching

	Group A	Group B	p-value
Age - Mean±SD	20.93±0.52	20.96±0.41	0.765
Gender - N (%)			
Male	11(36.7)	10(33.3)	0.787
Female	19(63.3)	20(66.7)	

Assessment scores

Table 2 shows the scores attained by the participants in each of the two groups in each of the two assessments. On comparing the scores of Group A and Group B participants in Assessment 1, no significant difference was noted. However, the scores in Assessment 2 showed a distinct significance in the outcomes of Group A compared to Group B. This was specifically noted in the scores obtained for the first two skills (Examination of tone and Elicitation of deep tendon reflexes) and in the overall score of Assessment 2.

**Table 2:** Comparison of the scores in Assessment 1 and 2, for Group A and B *shows statistically significant; SD = standard deviation Group A - participants were taught the clinical examination skills by the PFSA technique Group B - participants were taught the clinical examination skills by the CBT technique The differences in the means of the two groups A and B were assessed for statistical significance by the unpaired t-test. The level of significance was fixed at p<0.05 for statistical tests. Assessment 1 - OSCE assessment done on the next day after the clinical skills teaching session for both groups. Assessment 2 - OSCE assessment done after a week of the clinical skills teaching session for both groups. OSCE: Objective Structured Clinical Examination; PFSA: Peyton's four-step approach; CBT: conventional bedside teaching

	Mean±SD	Mean±SD	p-value
Assessment number 1	Group A	Group B	
Examination of tone	9.40±0.62	9.20±0.92	0.582
Elicitation of deep tendon reflexes	9.20±0.76	9.00±0.87	0.370
Elicitation of signs of free fluid in the abdomen	9.40±0.77	9.13±0.97	0.291
Total	28.00± 1.25	27.33±1.82	0.180
Assessment number 2	Group A	Group B	
Examination of tone	9.47±0.57	8.77±0.62	<0.001*
Elicitation of deep tendon reflexes	9.37±0.71	8.57±0.93	0.001*
Elicitation of signs of free fluid in the abdomen	8.90±0.96	8.50±0.82	0.070
Total	27.73±1.48	25.83±1.55	<0.001*

Outcome ratings (good performance rating)

Table 3 depicts the number of participants who obtained a good performance rating in each of the two assessments. Ninety percent of students in Group A and 63.3% of students in Group B secured a good performance rating in Assessment 1, while 83.3% of students in Group A and only 36.7 % of students in Group B secured a good performance rating in Assessment 2. On comparing the results of Group A with those of Group B, a significant difference was noted in both assessments with regard to the number of participants obtaining a good performance rating, with Group A scoring better. 

**Table 3:** Good performance rating *shows statistically significant Group A - participants were taught the clinical examination skills by the PFSA technique Group B - participants were taught the clinical examination skills by the CBT technique The differences in the outcomes of the two groups A and B were assessed for statistical significance by the unpaired t-test. The level of significance was fixed at p<0.05 for statistical tests. Assessment 1 - OSCE assessment done on the next day after the clinical skills teaching session for both groups. Assessment 2 - OSCE assessment done after a week of the clinical skills teaching session for both groups. Yes - Students had a Good Performance rating (27 or above out of 30) in OSCE assessments No - Students did not have a Good Performance rating (27 or above out of 30) in OSCE assessments OSCE: Objective Structured Clinical Examination; PFSA: Peyton's four-step approach; CBT: conventional bedside teaching

	Group A N (%)	Group B N (%)	p-value
Assessment 1			
Yes	27(90)	19(63.3)	0.015*
No	3(10)	11(36.7)	
Assessment 2			
Yes	25(83.3)	11(36.7)	<0.001*
No	5(16.7)	19(63.3)	

Perception responses

The results of the perception questionnaire were compared between the groups. Table 4 shows the inter-group comparison of responses for statistical significance.

**Table 4:** Inter-group comparison of responses in the perception questionnaire *shows statistically significant Group A - participants were taught the clinical examination skills by the PFSA technique Group B - participants were taught the clinical examination skills by the CBT technique The differences in the outcomes of the two groups A and B were assessed for statistical significance by the unpaired t-test. The level of significance was fixed at p<0.05 for statistical tests. PFSA: Peyton's four-step approach; CBT: conventional bedside teaching

Statements	Group A - N (%)	Group B - N (%)	p-value
1. The clinical demonstration was clear and lucid			
Agree	4(13.3)	19(63.3)	<0.001*
Strongly Agree	26(86.7)	11(36.7)	
2. The method improved my confidence in clinical examination			
Agree	9(30)	17(56.7)	0.037*
Strongly Agree	21(70)	13(43.3)	
3. Practical errors in clinical examination were clarified adequately			
Agree	2(6.7)	13(43.3)	0.001*
Strongly Agree	28(93.3)	17(56.7)	
4. The method enabled me to retain the clinical skills satisfactorily and correctly			
Agree	2(6.7)	13(43.3)	0.001*
Strongly Agree	28(93.3)	17(56.7)	
5. Overall satisfied by the clinical exposure and demonstration			
Agree	6(20)	24(80)	<0.001*
Strongly Agree	24(80)	6(20)	
6. Would like to learn more clinical skills by this method in future			
Agree	10(33.3)	15(50)	0.19
Strongly Agree	20(66.7)	15(50)	

## Discussion

This was an observational cross-sectional study to investigate and compare two different approaches, namely, CBT and PFSA, in teaching acquisition and retention of clinical examination skills. PFSA is an innovative and validated approach to convert learners from “consciously incompetent” to “consciously competent” states, allowing for mental representation and vocalization, along with observation and performance [22]. This study is a direct comparison of the PFSA technique versus the standard CBT method in imparting clinical examination skills. Students of Phase II MBBS were selected for this study and the study groups (A and B) did not differ in age or gender distribution.

It was noted that after the first Assessment, the scores of the participants of both the intervention group A and the control group B were similar, thus showing that the PFSA is as effective as CBT in helping students understand and acquire clinical examination skills. The similar efficacy of PFSA in teaching psychomotor skills has been explained in many recent studies [21-23].

However, in Assessment 2, which was a test for retention of the skills, the participants in Group A scored significantly better in two of the three skill sets, namely, examination of tone and elicitation of deep tendon reflexes. The overall score of Assessment 2, combining the scores of all three skill sets, was also found to be significantly superior in the PFSA group. Thus, PFSA brought about a measurable advantage in terms of skill retention in our study which showed consistency with other previous studies as well [9-12]. The results observed in the scores for the third skill, namely, elicitation of signs of free fluid in the abdomen, were nearly equivalent for both groups. These results emphasize that CBT remains a gold standard for teaching clinical skills, and innovative structured methods like PFSA can be creatively assimilated into teaching methodologies with similar or even superior results. Studies by Ramani et al. [17] and Rammell et al. [18] also reported similar results for teaching clinical skills in effective ways and methods. 

The surrogates of the primary and secondary outcomes of this study were the good performance ratings in both the assessments. The number of students getting a good performance rating in each of the assessments was significantly higher with Group A when compared to Group B. 

The perception questionnaire feedback results also strongly support the introduction of PFSA into medical and clinical teaching. There was not a single disagreement on any of the two teaching methodologies. The PFSA technique proved to be more popular and interesting for the students when compared to the perception responses for the conventional teaching method. The difference in responses was also found to be statistically significant in most of the questions posed. However, the results must be considered as subjective responses from students who were naïve to clinical or bedside teaching and need further objective corroboration. There is a large future scope for interventions in this prospect of medical education as there is a dearth of research in this as also reported by Schmidt et al. [19]. 

Along with credible and significant results, the study had a limited number of participants, belonging to a single phase of MBBS, studied over a short period and hence the results need to be substantiated with a larger sample, longer duration studies. Also, the results must be ideally compared with students of different phases of MBBS in order to get a comprehensive picture of skill learning in medical teaching. This was a single-center study and results may vary in different organizational or didactical settings. We did not have control over the self-study, motivation, external help, and other such factors that the participants may have had, and this could have influenced their scores in both assessments, which were conducted within a prefixed gap period. This study investigated the acquisition and retention of skills on a short-term and medium-term basis only. The study did not include an assessment of the retention of skills for a longer duration.

## Conclusions

PFSA has already been proven to be a good teaching method for the acquisition of complex procedural skills. This study expanded the role of PFSA in teaching clinical bedside examination skills to medical students. The results of this study show that PFSA is definitely suitable for teaching clinical examination skills. The acquisition of skills was found non-inferior to CBT while the retention of these skills was found to be equally good or even superior with PFSA than with CBT. Further large-scale studies may clarify the learning impact and outcomes of PFSA in CBT. This study could encourage further research in the field of innovative, scientific, and structured learning techniques for undergraduates in MBBS as well as other professional courses, especially clinical and paraclinical fields.
